# Hyperthermia enhances the efficacy of PD-1 inhibitor plus chemotherapy in PD-L1 CPS-negative advanced gastric cancer

**DOI:** 10.3389/fimmu.2026.1795363

**Published:** 2026-05-13

**Authors:** Weihang Li, Xin Sun, Ping Li, Ting Wang, Panling Xu

**Affiliations:** 1 Oncology Department of Integrated Traditional Chinese and Western Medicine, The First Affiliated Hospital of Anhui Medical University, Hefei, Anhui, China; 2 Department of Integrated Traditional Chinese and Western Medicine, Anhui Medical University, Hefei, Anhui, China

**Keywords:** circulating tumor cells, gastric cancer, hyperthermia, PD-1 inhibitor, programmed death-ligand 1

## Abstract

**Background:**

Advanced gastric cancer with negative PD-L1 combined positive score (CPS) responds poorly to immunotherapy. This study investigated the efficacy of hyperthermia combined with a PD-1 inhibitor and chemotherapy in this patient population.

**Methods:**

In this retrospective study, 72 patients with PD-L1 CPS-negative advanced gastric cancer were enrolled. 37 received hyperthermia plus PD-1 inhibitor and SOX chemotherapy, while 35 received PD-1 inhibitor and chemotherapy alone. Propensity score matching (PSM) was applied. Progression-free survival (PFS) was the primary endpoint. Adverse events and circulating tumor cell dynamics were also assessed.

**Results:**

After PSM, baseline characteristics were well-balanced. The CIH group showed significantly prolonged PFS compared to the CI group, both before and after matching (P = 0.00037 and P = 0.0011, respectively). The CIH group also had a significantly lower incidence of fatigue and anorexia (P<0.05). Although circulating tumor cell (CTC) counts increased in both groups after treatment, the post-treatment count and the magnitude of increase were significantly greater in the CIH group (P<0.001), potentially indicating enhanced therapy-induced immune activation and tumor cell death.

**Conclusion:**

The addition of hyperthermia to PD-1 inhibitor and chemotherapy significantly improves PFS and reduces specific adverse events in patients with PD-L1 CPS-negative advanced GC. This distinct CTC dynamic change, although still requiring further verification, may offer a potential mechanistic insight into the significant efficacy of this combined strategy.

## 1 Introduction

The incidence of gastric cancer is high, ranking as the 5th most common malignant tumor worldwide (1). Most patients are diagnosed at an advanced stage, with a poor prognosis (2). In recent years, the emergence of immunotherapy has changed the treatment strategy for advanced gastric cancer. According to the Checkmate 649 clinical study (3), for patients with advanced gastric cancer who are PD-L1 CPS > 5 and HER2-negative, the median progression-free survival of the first-line treatment using nivolumab + chemotherapy group compared to the chemotherapy group nearly doubled, being 8.5 months and 4.3 months respectively (HR 0.51); however, for patients with PD-L1 CPS negative, the addition of immunotherapy did not prolong the survival time of the patients. Based on this, the treatment plan for this group of patients urgently needs improvement.

Hyperthermia is achieved by selectively increasing the temperature of tumor tissue through physical energy and utilizing its biological effects to enhance anti-tumor effects (4). Studies have shown that hyperthermia can induce tumor cell immunogenic death, release damage-associated molecular patterns (DAMPs), activate dendritic cells and promote T-cell-mediated immune responses (5, 6). Moreover, hyperthermia can improve the tumor microenvironment, increase immune cell infiltration and reduce the secretion of immunosuppressive factors, thereby synergistically enhancing the efficacy of chemotherapy and immunotherapy (7), Notably, hyperthermia can activate the NF-κB pathway by upregulating heat shock proteins (HSPs), promoting PD-L1 expression in tumor cells (8), providing a potential mechanism for enhancing the immunotherapy efficacy in PD-L1-negative patients. Although hyperthermia has demonstrated clinical value in breast cancer, brain tumors, etc. (9, 10), its synergistic effect in PD-L1 CPS-negative gastric cancer still needs further verification.

Hyperthermia promotes tumor antigen presentation, activates DC cells to secrete IL-1β (11), and enhances the killing function of CD8^+^ T cells to activate the immune system. Local hyperthermia increases tumor blood flow (12),improves the hypoxic/acidic microenvironment, promotes the infiltration of T cells and NK cells, and inhibits the activity of Treg/MDSCs and the secretion of TGF-β/IL-10, reshaping the immune microenvironment. These mechanisms make hyperthermia a potential tumor treatment method, especially when combined with other therapies (13). PD-L1 CPS-negative patients (CPS < 1) have limited efficacy in immunotherapy, especially in gastric cancer and other tumor types, where the response rate of PD-1/PD-L1 inhibitors alone is relatively low (14, 15). This study aims to explore the efficacy and mechanism of hyperthermia combined with PD-1 inhibitors and chemotherapy in PD-L1 CPS-negative advanced gastric cancer patients through a retrospective analysis, providing a basis for optimizing the treatment strategy for this group of patients.

## 2 Materials and methods

### 2.1 Study design and participants

This study is a retrospective clinical research aimed at evaluating the efficacy and safety of hyperthermia combined with PD-1 inhibitors and chemotherapy in patients with advanced gastric cancer who have PD-L1 CPS negativity and HER2 negativity. The research data were derived from patients with advanced gastric cancer who received treatment at the Oncology Center of the First Affiliated Hospital of Anhui Medical University from January 1, 2021 to June 30, 2022. A total of 103 patients who received hyperthermia combined with first-line treatment and 101 patients who only received first-line treatment were screened. Eventually, 72 patients were included, among whom 37 received chemotherapy, PD-1 inhibitor combined with hyperthermia (CIH group) and 35 received chemotherapy combined with PD-1 inhibitors (CI group). The group allocation for hyperthermia was determined through joint decision-making by doctors and patients, based on the patient’s physical condition (ECOG score of 0-1), informed consent, and availability of equipment. Those who did not receive hyperthermia included those who refused, were intolerant, or were unable to cooperate.

This study enrolled patients with pathologically diagnosed advanced gastric adenocarcinoma, PD-L1 CPS and HER2 negative, who received PD-1 inhibitor combined with SOX regimen as first-line treatment and were followed until disease progression. Exclusion criteria were nonadenocarcinoma histologic type, PD-L1 CPS or HER2 positivity, treatment discontinuation because of immune-related adverse events, incomplete clinical or follow-up data, and use of other or non-first-line treatment options.

### 2.2 Treatment regimens

All patients received PD-1 inhibitor (Sintilimab Injection) combined with SOX chemotherapy (oxaliplatin + tegafur, with tegafur administered at 40 mg twice daily for 14 consecutive days). The dosage and frequency of PD-1 inhibitor use were 200 mg every 3 weeks.

Patients in the hyperthermia group received deep hyperthermia in addition to chemotherapy and immunotherapy. The hyperthermia equipment was NW-9001 Microwave Tumor Hyperthermia Device, with a temperature of 40.5°C. Each session lasted for 40 minutes, and a cycle consisted of 5 consecutive days (16, 17). This cycle was repeated every 21 days. The hyperthermia area was the primary or metastatic lesion in the stomach (such as liver metastasis, peritoneal metastasis, etc.), until disease progression or intolerable adverse reactions occurred. During the treatment process, standardized control is carried out through the feedback of the patient’s body surface temperature and the preset parameters of the equipment.

All patients received standard supportive care according to institutional guidelines, including antiemetics, nutritional support, and pain management as needed. There were no systematic differences in supportive care protocols between the two groups, as the decision to add hyperthermia did not alter the standard supportive care regimen.

### 2.3 Outcomes and definitions

This study defined PFS as the primary endpoint, from the start of treatment to disease progression or death from any cause. In addition, treatment-related adverse events (AEs) were evaluated according to the CTCAE v5.0 standard, and the dynamic changes of amplified circulating tumor cells (CTCs) before and after treatment.

### 2.4 Data collection and statistical analysis

This study collected data on patients’ baseline characteristics, PFS, AEs, and CTCs. To control for confounding bias, propensity score matching (PSM) was used to perform 1:1 matching between the two groups of patients. Matching variables included key baseline indicators such as age, gender, and tumor location. After matching, comparisons of categorical and continuous variables between the groups were conducted using the χ 2 test and t-test/Wilcoxon test, respectively. PFS analysis was performed using the Kaplan-Meier (K-M) method and Log-rank test. For each type of adverse event occurrence, 2×2 contingency tables were constructed respectively. The appropriate statistical method was selected based on the expected frequency criterion (Pearson chi-square test was used when all expected frequencies were ≥ 5, otherwise Fisher’s exact probability method was used) for the comparison between groups. Comparisons of CTC within and between groups were conducted using paired t-test and independent sample t-test/Mann-Whitney U test. The analysis was conducted using R software (v4.2.2), and differences were considered statistically significant if P < 0.05.

### 2.5 Ethics and informed consent

This study has been approved by the Ethics Committee of the First Affiliated Hospital of Anhui Medical University (Ethics Number: NO. PJ2024-05-74). As this study is a retrospective study, the patients’ informed consent was waived, but all the patients’ personal information has been anonymized to protect their privacy.

## 3 Results

### 3.1 Patient characteristics

**Figure 1:**
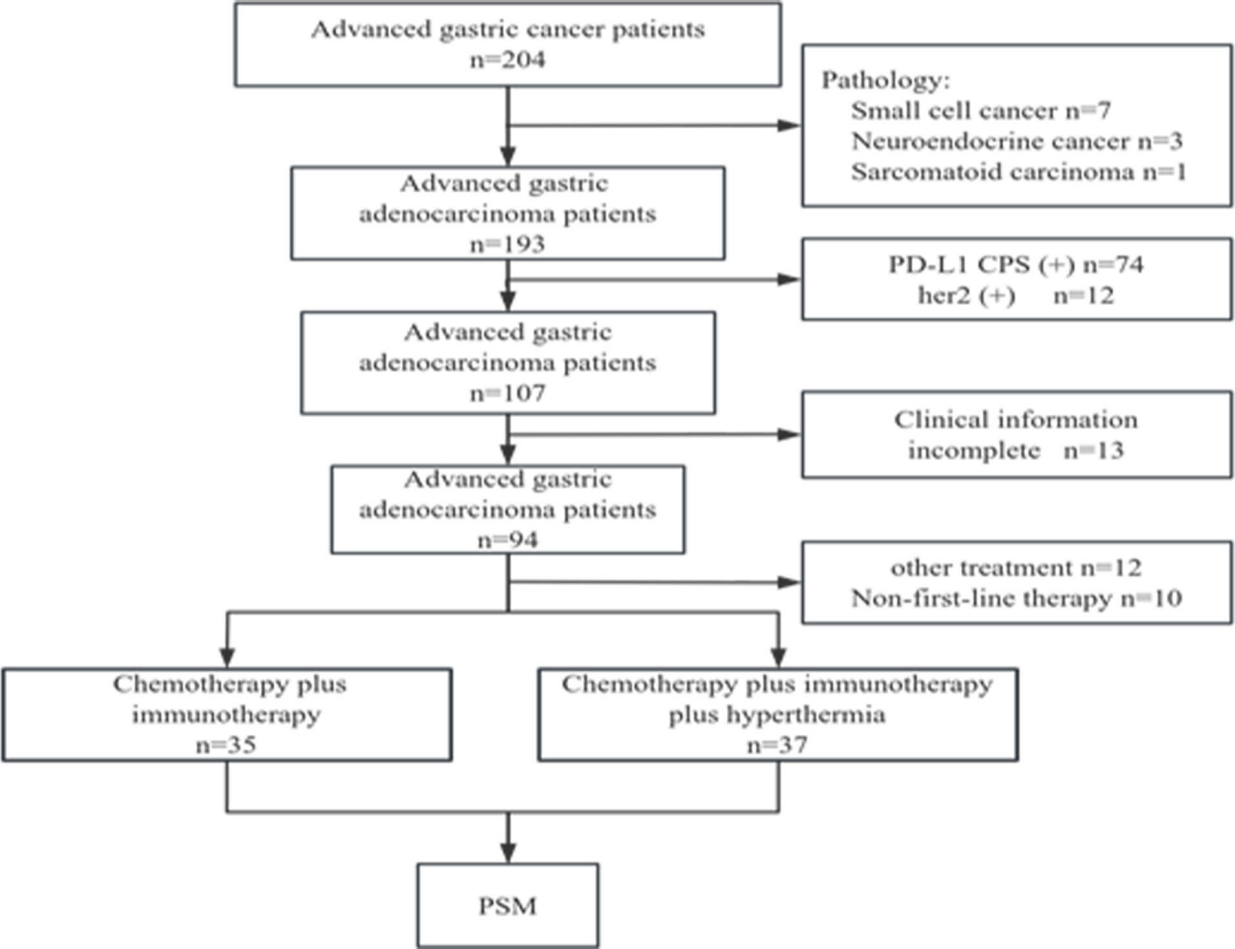
Patient screening flow chart.

We screened 204 patients with advanced gastric cancer and excluded those with non-adenocarcinoma (n=11), PD-L1 CPS (+) (n=74), Her-2 (+) (n=12), incomplete clinical information (n=13), other treatment regimens, and non-first-line treatment regimens (n=22). Finally, a total of 72 patients with PD-L1 CPS-negative advanced gastric cancer were included. Among them, 35 received first-line treatment (CI), and 37 received first-line treatment combined with hyperthermia (CIH) (**Figure 1**). After PSM, 52 patients (26 in each group) were retained for comparative analysis.

Among them, in the pre-matching queue, the median age distribution of the CI group (n=35) and the CIH group (n=37) was similar, and there was no significant difference among different age categories (P = 0.996).754; AFP: P = 0.934) were also balanced (**Table 1**).

**Table 1:** The baseline characteristics of 72 patients.

Covariates	Type	All(n=72) n (%)	CI(n=35) n (%)	CIH(n=37) n (%)	P-value
Age (year)	< 45	4 (5.6%)	2 (5.7%)	2 (5.4%)	0.996
	45 ~ 70	43 (59.7%)	21 (60.0%)	22 (59.5%)	
	>70	25 (34.7%)	12 (34.3%)	13 (35.1%)	
Sex	Female	25 (34.7%)	11 (31.4%)	14 (37.8%)	0.934
	Male	47 (65.3%)	24 (68.6%)	23 (62.2%)	
Primary tumor location	Gastric	24 (33.3%)	12 (34.3%)	12 (32.4%)	0.957
	cardia	20 (27.8%)	10 (28.6%)	10 (27.1%)	
	Antrum	28 (38.9%)	13 (37.1%)	15 (40.5%)	
Tumor differentiation	Poor	64 (88.9%)	31 (88.6%)	33 (89.2%)	>0.999
	Moderate	8 (11.1%)	4 (11.4%)	4 (10.8%)	
	High	0 (0.0%)	0 (0.0%)	0 (0.0%)	
Liver metastasis	Yes	38 (52.8%)	18 (51.4%)	20 (54.1%)	0.824
	No	34 (47.2%)	17 (48.6%)	17 (45.9%)	
CEA	< 5	46 (63.9%)	23 (65.7%)	23 (62.2%)	0.754
	5	26 (36.1%)	12 (34.3%)	14 (37.8%)	
AFP	< 8.1	64 (88.9%)	31 (88.6%)	33 (89.2%)	0.934
	8.1	8 (11.1%)	4 (11.4%)	4 (10.8%)	

After PSM, there were 26 cases in each group. The age and gender distribution remained balanced (Age: P = 0.965; SEX: P = 0.445); the distribution of tumor locations was uniform among all groups (P = 0.957); there was no difference in the degree of differentiation between the two groups (P = 0.873). There were no significant differences in the rate of liver metastasis and the distribution of biomarkers (Liver metastasis: P = 0.820; CEA: P = 0.649; AFP: P = 0.844). (**Table 2**).

**Table 2:** After performing propensity score matching (PSM) on the patients, a total of 26 patients in each group (26 vs 26) were included in the baseline table.

Covariates	Type	All (n=52) n (%)	CI (n=26) n (%)	CIH(n=26) n (%)	P-value
Age (year)	< 45	3 (5.8%)	2 (7.7%)	1 (3.8%)	0.965
	45 ~ 70	31 (59.6%)	16 (61.5%)	15 (57.7%)	
	>70	18 (34.6%)	8 (30.8%)	10 (38.5%)	
Sex	Female	17 (32.7%)	9 (34.6%)	8 (30.8%)	0.445
	Male	35 (67.3%)	17 (65.4%)	18 (69.2%)	
Primary tumor location	Gastric	16 (30.8%)	8 (30.8%)	8 (30.8%)	0.740
	cardia	16 (30.8%)	7 (26.9%)	9 (34.6%)	
	Antrum	20 (38.4%)	11 (42.3%)	9 (34.6%)	
Tumor differentiation	Poor	48 (92.3%)	24 (92.3%)	24 (92.3%)	0.873
	Moderate	4 (7.7%)	2 (7.7%)	2 (7.7%)	
	High	0 (0.0%)	0 (0.0%)	0 (0.0%)	
Liver metastasis	Yes	26 (50.0%)	12 (46.2%)	14 (53.8%)	0.820
	No	26 (50.0%)	14 (53.8%)	12 (46.2%)	
CEA	< 5	33 (63.5%)	18 (69.2%)	15 (57.7%)	0.649
	5	19 (36.5%)	8 (30.8%)	11 (42.3%)	
AFP	< 8.1	45 (86.5%)	22 (84.6%)	23 (88.5%)	0.844
	8.1	7 (13.5%)	4 (15.4%)	3 (11.5%)	

These results confirm that the baseline characteristics of the CI group and the CIH group were well balanced before and after pairing, supporting the validity of the subsequent efficacy comparison analysis.

### 3.2 Evaluation of the effectiveness of the treatment plan

K-M analysis was conducted separately for the two groups of patients before and after PSM.

**Figure 2:**
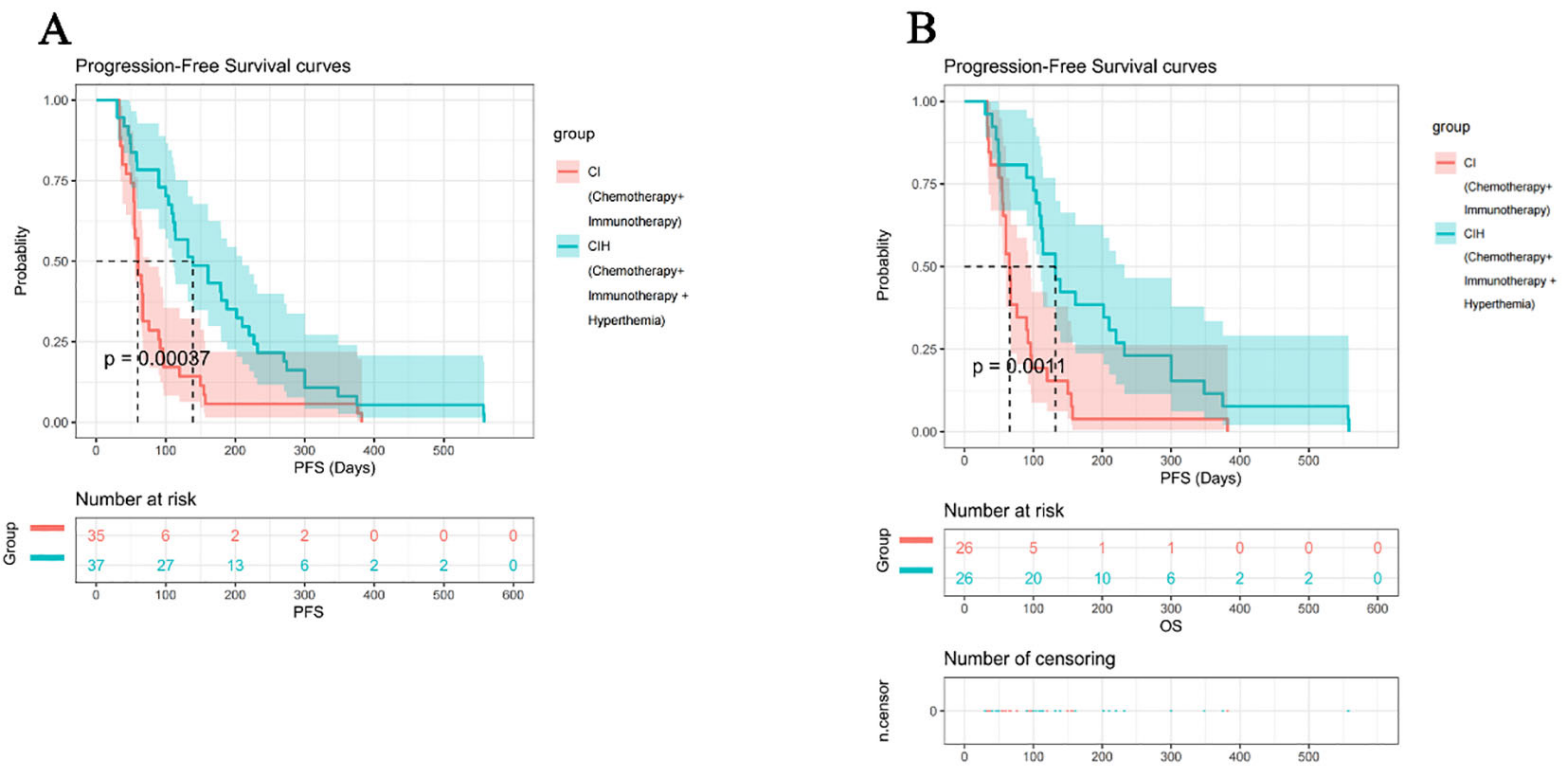
Analysis of PFS between the two groups. **(A)** Pre-Matching Cohort; **(B)** Post-Matching Cohort. The CIH group exhibited significantly prolonged PFS compared to the CI group, both before and after PSM.

Before PSM, the median progression-free survival (PFS) was significantly longer in the CIH group (n=37) than in the CI group (n=35) [139 days (95% CI 111–210) vs. 60 days (95% CI 55–76); hazard ratio (HR) for disease progression or death in the CIH group relative to the CI group = 0.42 (95% CI: 0.26–0.68; log-rank P = 0.00037)] (**Figure 2A**). As time progressed, the number of patients at risk in both groups gradually decreased. The K-M curve analysis showed a significant difference in PFS between the CI group and the CIH group. At 300 days, the number of patients at risk in the CI group decreased sharply (from 35 to 2), while the proportion of patients at risk in the CIH group was higher (from 37 to 13). After more than 500 days, no patients in the CI group remained at risk, while 2 patients remained at risk in the CIH group. These results indicate that in the pre-matched cohort, the CIH regimen has a significant survival advantage.

After PSM, the PFS benefit of CIH remained significant, with a median PFS of 132 days (95% CI 109–232) in the CIH group versus 65.5 days (95% CI 56–96) in the CI group (HR = 0.39, 95% CI: 0.22–0.70; log-rank P = 0.0011) (**Figure 2B**).At 300 days, the number of patients at risk in the CI group decreased from 26 to 1, while the number of patients at risk in the CIH group remained at a relatively high level (from 26 to 6). By 500 days, both groups had the fewest number of at-risk patients (CI: 0 vs. CIH: 2), further confirming the durability of the therapeutic effect of CIH. The consistency of the improvement in PFS before and after matching emphasizes the robustness of hyperthermia as an adjuvant treatment.

This means that adding hyperthermia to chemotherapy and PD-1 inhibition can significantly improve the PFS of patients with PD-L1 CPS-negative advanced gastric cancer.

### 3.3 Statistics of adverse reactions

By conducting a statistical and analytical study on the adverse events in the post-PSM matching cohort, it can be observed that compared with the CIH group, the incidence of fatigue and anorexia in the CI group was significantly higher (P < 0.05). Specifically, 34.6% of the CI patients experienced fatigue, while 11.5% of the CIH patients experienced anorexia, and 50.0% of the CI group and 23.1% of the CIH group experienced anorexia. Other adverse events included diarrhea, fever, nausea, and vomiting, and there was no statistically significant difference between the groups. The addition of hyperthermia therapy on the basis of chemotherapy and immunotherapy was associated with a reduction in the incidence of fatigue and anorexia. Most adverse reactions (such as diarrhea and nausea) were comparable between the two groups, indicating that hyperthermia therapy did not have an additional toxic burden. The CIH regimen not only improved efficacy (as shown in the PFS analysis) but also demonstrated good safety, especially in reducing fatigue and anorexia. The CIH treatment regimen has high safety.

### 3.4 Analysis of circulating tumor cells results

**Figure 3:**
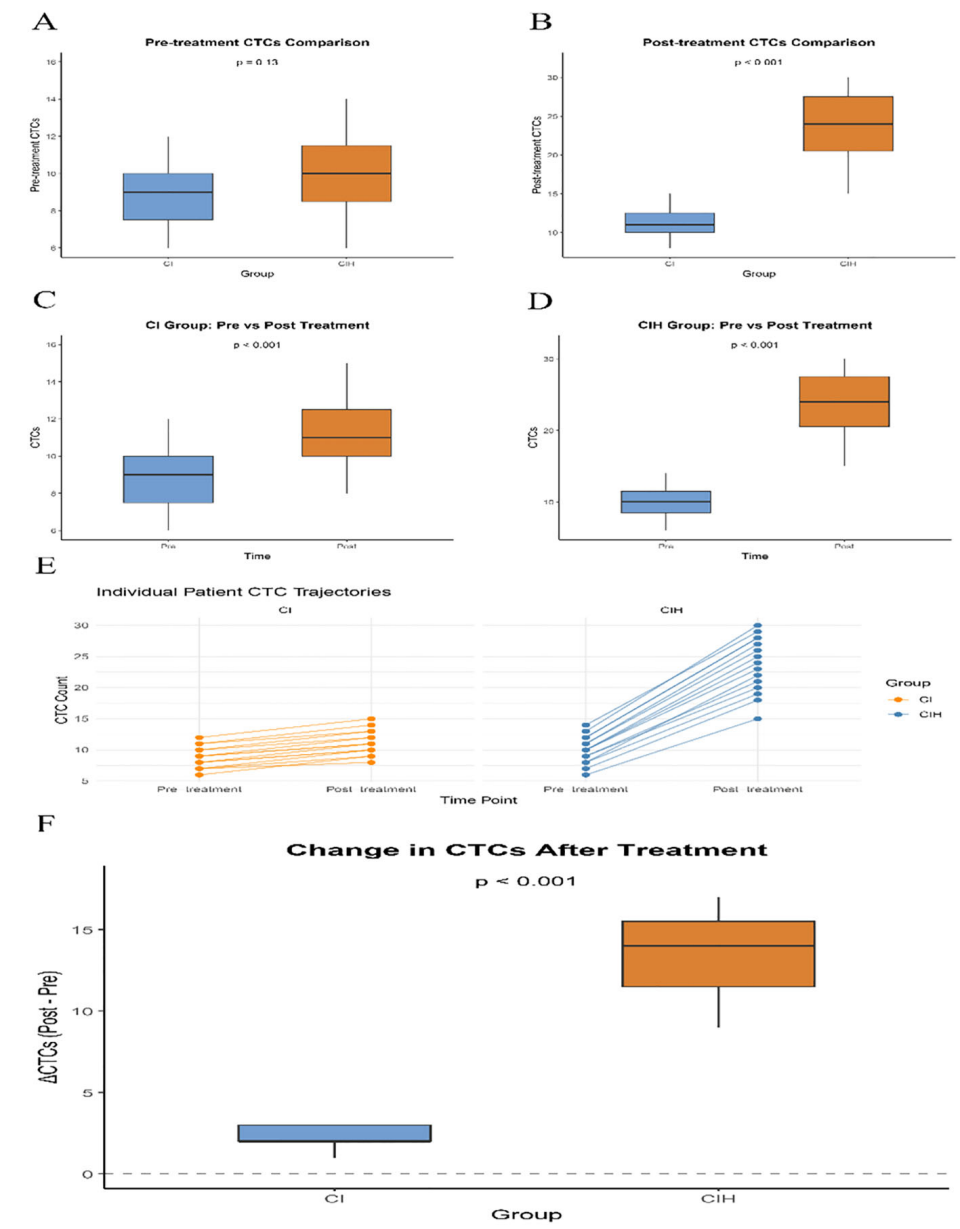
Patient CTCs analysis chart. **(A)** Pre-treatment CTCs Comparison; **(B)** Post-treatment CTCs Comparison; **(C)** Comparison of CTCs in CI group before and after treatment; **(D)** Comparison of CTCs in CIH group before and after treatment; **(E)** Individual patient trajectories of CTC counts from pre- to post-treatment; **(F)** Comparison of CTCs change amplitude before and after treatment between two groups.

To deeply explore the biological effects of hyperthermia, both groups of patients underwent the detection of CTCs before and after treatment. We longitudinally monitored the dynamic changes of CTCs in patients. The results showed that there was no significant difference in the baseline CTCs levels between the two groups of patients before treatment (**Figure 3A**, P = 0.13), ensuring the comparability of the initial state. After the treatment, there was a significant difference in the number of CTCs between the two groups (**Figure 3B**, P < 0.001). The intra-group analysis revealed that the trends of CTCs numbers in the two groups were similar. After treatment, the CTC count in the CI group increased compared to the baseline value (**Figure 3C**; P < 0.001), and correspondingly, the CTC count in the CIH group also increased compared to the baseline (**Figure 3D**; P < 0.001). The CTC trajectories of individual patients (**Figure 3E**) clearly illustrate the distinct change patterns of the two groups: In the CIH group, the CTC counts of most patients showed a steeper upward trajectory, indicating that the addition of hyperthermia was associated with significant fluctuations in CTC levels. To precisely quantify this change, we calculated the change in CTCs (ΔCTCs) before and after treatment for the patients. We found that the increase in CTCs in the CIH group was significantly greater than that in the CI group (**Figure 3F**, P < 0.001).

## 4 Discussion

This retrospective study provides compelling evidence that the addition of hyperthermia to chemotherapy and PD-1 inhibitor therapy can significantly improve the PFS of patients with PD-L1 CPS-negative advanced gastric cancer (GC), while reducing the incidence of treatment-related fatigue and anorexia. The consistency of the study results before and after PSM highlights the robustness of the therapeutic benefits brought by hyperthermia, indicating its potential as an adjuvant treatment in this patient population with a high treatment difficulty.

The observed PFS advantage in the CIH group is consistent with emerging evidence, suggesting that hyperthermia may exert a synergistic effect with immunotherapy by modulating the tumor microenvironment (TME). It is traditionally believed that PD-L1 CPS-negative tumors have reduced antigen presentation and immune infiltration, resulting in a poorer response to immune checkpoint inhibitors (ICIs) (18, 19). However, hyperthermia has been proven to enhance tumor immunogenicity through the upregulation of heat-inducible stress proteins (such as HSP70) and the expression of major histocompatibility complex class I (8, 20). Additionally, local hyperthermia may promote lymphocyte migration within the tumor and the activation of dendritic cells (5), potentially overcoming the inherent immune inertia of PD-L1-negative tumors. This immune regulatory effect can explain the phenomenon that patients treated with CIH still achieve sustained survival benefits even in populations that are typically resistant to PD-1/PD-L1 blockade.

This study observed that the levels of circulating tumor cells (CTCs) in both groups significantly increased after treatment, and the increase was more pronounced in the CIH group. This phenomenon seems contradictory at first glance: an increase in CTCs is typically associated with a poor prognosis in solid tumors (21) yet the CIH group showed an improvement in progression-free survival (PFS). However, in the context of combined treatment, this finding should be interpreted with caution. This observation result may be caused by multiple mechanisms. One possibility is that cytotoxic intervention measures (including chemotherapy and immune therapies that bind to T cells) can damage the integrity of the tumor (22), thereby causing tumor cells to temporarily release into the bloodstream during the treatment process. Another possible factor is hyperthermia, which has been proven to induce immunogenic cell death and promote the release of tumor antigens (23); this process can also potentially lead to a temporary increase in detectable circulating tumor cell (CTC) levels. Therefore, the observed surge in CTC numbers may reflect the active breakdown of the tumor and the immune response, rather than indicating an indicator of the progression of metastatic disease.

The reduced incidence of fatigue and anorexia in the CIH group provides a new perspective on the safety of hyperthermia. Although these symptoms are typically attributed to the toxic effects of chemotherapy and immunotherapy (24), the improvement of these symptoms by hyperthermia may reflect an improvement in metabolic regulation or a reduction in systemic inflammation. However, further studies are needed to distinguish these effects from possible confounding factors, such as differences in supportive treatment measures between the treatment groups.

This study has several limitations. First, the retrospective design carries inherent selection bias, while propensity score matching (PSM) balanced observed confounders, residual confounding may persist. Second, the limited sample size was further reduced after PSM, constraining result robustness. Third, our CTC assay lacked assessment of cell viability, apoptotic status, and phenotypic markers (e.g., epithelial-mesenchymal transition), precluding discrimination between metastasis-competent viable cells and inactive/immune-coated cells of treatment-related origin. Additionally, direct correlation between CTC kinetics and individual outcomes was not established, leaving prognostic utility uncertain. Accordingly, the observed intergroup CTC response difference should be interpreted as exploratory, not conclusive. Future studies incorporating multidimensional CTC characterization (viability assays, phenotyping, single-cell analysis) are warranted to clarify whether CTC elevations reflect enhanced treatment response, transient mechanical shedding, or both. Finally, the impact of hyperthermia parameters (temperature, duration, frequency) on therapeutic efficacy requires further investigation for protocol optimization.

The results of this study indicate that hyperthermia combined with PD-1 inhibitors and chemotherapy has potential clinical value in patients with PD-L1 CPS-negative advanced gastric cancer. Hyperthermia, as a non-invasive and less side-effect treatment method, can be used as an effective adjuvant treatment strategy, especially in patients where the efficacy of single-agent immunotherapy is limited. Future research can further explore the combination of hyperthermia with other immunotherapy drugs (such as CTLA-4 inhibitors, LAG-3 inhibitors, etc.) and the wide applicability of hyperthermia in different tumor types.

## 5 Conclusion

Adding hyperthermia to the regimen of PD-1 inhibitors combined with chemotherapy can significantly prolong the PFS of patients with PD-L1 CPS-negative advanced gastric cancer, and also shows good safety. This distinct CTC dynamic change, although still requiring further verification, may offer a potential mechanistic insight into the significant efficacy of this combined strategy.

## Data Availability

The original contributions presented in the study are included in the article/supplementary material. Further inquiries can be directed to the corresponding authors.

## References

[1] Zheng Y Qiu B Liu S Song R Yang X Wu L . A transformer-based deep learning model for early prediction of lymph node metastasis in locally advanced gastric cancer after neoadjuvant chemotherapy using pretreatment CT images. EClinicalMedicine. (2024) 75:102805. doi: 10.1016/j.eclinm.2024.102805. PMID: 39281097 PMC11402411

[2] Sun Q Li S Lou J Wang X Xu X . Recent advances in tumour microenvironment impact immunotherapy resistance in gastric cancer. Crit Rev Oncol Hematol. (2025) 215:104837. doi: 10.1016/j.critrevonc.2025.104837. PMID: 40618861

[3] Janjigian YY Shitara K Moehler M Garrido M Salman P Shen L . First-line nivolumab plus chemotherapy versus chemotherapy alone for advanced gastric, gastro-oesophageal junction, and oesophageal adenocarcinoma (CheckMate 649): a randomised, open-label, phase 3 trial. Lancet. (2021) 398:27–40. doi: 10.1016/s0140-6736(21)00797-2. PMID: 34102137 PMC8436782

[4] McGrath K Frain M Hey G Rahman M . Complications following laser interstitial thermal therapy: a review. Neurochirurgie. (2025) 71:101604. doi: 10.1016/j.neuchi.2024.101604. PMID: 39413572

[5] Abreu MM Chocron AF Smadja DM . From cold to hot: mechanisms of hyperthermia in modulating tumor immunology for enhanced immunotherapy. Front Immunol. (2025) 16:1487296. doi: 10.3389/fimmu.2025.1487296. PMID: 40092992 PMC11906415

[6] Fei C Zhen X Shiqiang Z Jun P . Frontier knowledge and future directions of programmed cell death in clear cell renal cell carcinoma. Cell Death Discov. (2024) 10:113. doi: 10.1038/s41420-024-01880-0. PMID: 38443363 PMC10914743

[7] Li K Feng J Deng J Shi H Peng R Zhang X . Hyperthermia inhibits the progression of gastric cancer by downregulating PLEK2/PD-L1 and possibly participates in immunomodulation. Gene. (2024) 898:148111. doi: 10.1016/j.gene.2023.148111. PMID: 38147897

[8] Yang Z Gao D Zhao J Yang G Guo M Wang Y . Thermal immuno-nanomedicine in cancer. Nat Rev Clin Oncol. (2023) 20:116–34. doi: 10.1038/s41571-022-00717-y. PMID: 36604531

[9] Zhu M Yang M Zhang J Yin Y Fan X Zhang Y . Immunogenic cell death induction by ionizing radiation. Front Immunol. (2021) 12:705361. doi: 10.3389/fimmu.2021.705361. PMID: 34489957 PMC8417736

[10] Fang H Ang B Xu X Huang X Wu Y Sun Y . TLR4 is essential for dendritic cell activation and anti-tumor T-cell response enhancement by DAMPs released from chemically stressed cancer cells. Cell Mol Immunol. (2014) 11:150–9. doi: 10.1038/cmi.2013.59. PMID: 24362470 PMC4003380

[11] de Lima JD de Paula AGP Yuasa BS de Souza Smanioto CC da Cruz Silva MC Dos Santos PI . Genetic and epigenetic regulation of the innate immune response to gout. Immunol Invest. (2023) 52:364–97. doi: 10.1080/08820139.2023.2168554. PMID: 36745138

[12] Vaupel P Piazena H Notter M Thomsen AR Grosu AL Scholkmann F . From localized mild hyperthermia to improved tumor oxygenation: physiological mechanisms critically involved in oncologic thermo-radio-immunotherapy. Cancers (Basel). (2023) 15(5):1394. doi: 10.3390/cancers15051394. PMID: 36900190 PMC10000497

[13] Zhang X Du Y Qin L Li B Wu Q Meng X . Synergistic microwave hyperthermia treatment for subcutaneous deep in situ breast cancer using conformal array antennas and a microwave-thermal-sensitive nanomaterial. J Mater Chem B. (2025) 13:524–35. doi: 10.1039/d4tb02319f. PMID: 39601758

[14] Janjigian YY Kawazoe A Bai Y Xu J Lonardi S Metges JP . 1400O Final overall survival for the phase III, KEYNOTE-811 study of pembrolizumab plus trastuzumab and chemotherapy for HER2+ advanced, unresectable or metastatic G/GEJ adenocarcinoma. Ann Oncol. (2024) 35:S877–S8. doi: 10.1016/j.annonc.2024.08.1466. PMID: 38826717

[15] Nytko KJ Weyland MS Dressel-Böhm S Scheidegger S Salvermoser L Werner C . Extracellular heat shock protein 70 levels in tumour-bearing dogs and cats treated with radiation therapy and hyperthermia. Vet Comp Oncol. (2023) 21:605–15. doi: 10.1111/vco.12923. PMID: 37653682

[16] Ademaj A Veltsista DP Ghadjar P Marder D Oberacker E Ott OJ . Clinical evidence for thermometric parameters to guide hyperthermia treatment. Cancers (Basel). (2022) 14(3):625. doi: 10.3390/cancers14030625. PMID: 35158893 PMC8833668

[17] Ren M Xie J Liu J Wang Y Xiang Z Li S . The clinical significance of hyperthermic intraperitoneal chemotherapy combined with PD-1 inhibitor and systemic chemotherapy for advanced gastric cancer patients with peritoneal metastasis: a single-center retrospective study. Front Oncol. (2025) 15:1728724. doi: 10.3389/fonc.2025.1728724. PMID: 41602425 PMC12832277

[18] Bai Y Yang W Käsmann L Sorich MJ Tao H Hu Y . Immunotherapy for advanced non-small cell lung cancer with negative programmed death-ligand 1 expression: a literature review. Transl Lung Cancer Res. (2024) 13:398–422. doi: 10.21037/tlcr-23-144. PMID: 38496691 PMC10938091

[19] Tong G Zhu M Chen Y Wang S Cheng B Wang S . Intratumoral CD8(+) T cells as a potential positive predictor of chemoimmunotherapy response in PD-L1-negative advanced gastric cancer patients: a retrospective cohort study. J Gastrointest Oncol. (2022) 13:1668–78. doi: 10.21037/jgo-22-644. PMID: 36092315 PMC9459198

[20] Wu B Zhang B Li B Wu H Jiang M . Cold and hot tumors: from molecular mechanisms to targeted therapy. Signal Transduct Target Ther. (2024) 9:274. doi: 10.1038/s41392-024-01979-x. PMID: 39420203 PMC11491057

[21] Siringo M Meo M Gelibter AJ Nicolazzo C Gazzaniga P . Prognostic value of circulating tumor cells and cancer associated macrophage-like cells in metastatic non-small cell lung cancer patients: a retrospective exploratory analysis. Oncol Res. (2026) 34:11. doi: 10.32604/or.2025.069832. PMID: 41613790 PMC12848706

[22] Feng Y Liu G Cai Q Zhou J . Unveiling the dual role of circulating tumor cells in colorectal cancer immunotherapy: a comprehensive review of biomarker utility and immune microenvironment crosstalk. Front Immunol. (2025) 16:1591359. doi: 10.3389/fimmu.2025.1591359. PMID: 40547026 PMC12179166

[23] Kennedy BE Noftall EB Dean C Roth A Clark KN Rowles D . Targeted intra-tumoral hyperthermia using uniquely biocompatible gold nanorods induces strong immunogenic cell death in two immunogenically 'cold' tumor models. Front Immunol. (2024) 15:1512543. doi: 10.3389/fimmu.2024.1512543. PMID: 39872527 PMC11769938

[24] Takahashi S Matsumoto K Ohba K Nakano Y Miyazawa Y Kawaguchi T . The incidence and management of cancer-related anorexia during treatment with vascular endothelial growth factor receptor-tyrosine kinase inhibitors. Cancer Manag Res. (2023) 15:1033–46. doi: 10.2147/cmar.s417238. PMID: 37771675 PMC10522463

